# Family physician deployment in South Africa’s district health system: A cross-sectional study

**DOI:** 10.4102/safp.v68i1.6289

**Published:** 2026-05-15

**Authors:** Theresia A. Rubler, Klaus B. von Pressentin, Robert J. Mash

**Affiliations:** 1 Division of Family Medicine, Department of Family, Community and Emergency Care, Faculty of Health Sciences, University of Cape Town, Cape Town, South Africa; 2 Division of Family Medicine and Primary Care, Faculty of Medicine and Health Sciences, Stellenbosch University, Cape Town, South Africa

**Keywords:** family physicians, family medicine, district health system, Primary Health Care universal health coverage, human resources for health, South Africa, specialist deployment

## Abstract

**Background:**

Family physicians (FP) strengthen the quality of district health services. The South African Academy of Family Physicians (SAAFP) recommended that, by 2030, there be one FP per district hospital, community health centre or subdistrict without a community health centre. This study aimed to evaluate progress towards achieving the SAAFP’s goals.

**Methods:**

A cross-sectional, descriptive survey collected data from senior academics in family medicine at all ten universities for 2024. Data focused on the presence, number and characteristics of employment of FPs across all 52 districts, 242 subdistricts and 607 district health facilities. Descriptive and mixed-effects regression analyses were used.

**Results:**

South Africa achieved 18.9% of the SAAFP goals, with enormous variability between provinces. Gauteng achieved 55% coverage, while the Northern Cape had no coverage. The Western Cape (34.5%) and Limpopo (23.4%) exceeded that national average. Family physicians were more likely to be employed in district hospitals (odds ratio [OR] 8.5; 95% confidence interval [CI]: 4.1–17.4, *p* < 0.001) and metropolitan districts (OR 20.9; 95% CI: 4.0–109.4, *p* < 0.001). Approximately 1 in 10 held medical officer posts (11.7%), and almost all were employed full time (96.3%). A substantial number (40.7%) were joint staff with universities.

**Conclusion:**

The deployment of FPs within the district health system has expanded but remains limited relative to SAAFP goals, with ongoing inequities in distribution. Achieving the goals is possible with the commitment of national and provincial policymakers and of human resources for health planners.

**Contribution:**

This study provides the first national mapping of FP distribution in the district health system, offering critical insights for workforce planning and policy implementation in support of universal health coverage.

## Introduction

Achieving equitable, accessible and quality healthcare for all South Africans remains a national priority, as enshrined in the Constitution (Section 27[1][a]) and aligned with the global Sustainable Development Goals (SDGs).^1,2^ Despite significant policy commitments, the South African health system continues to face the challenges of persistent communicable diseases (e.g. human immunodeficiency virus [HIV] and tuberculosis), an increasing incidence of non-communicable diseases (e.g. diabetes and cardiovascular conditions), maternal and child mortality and high rates of injury and trauma.^3,4^ The burden of disease is fuelled by underlying poverty, inequality and unemployment.^4^ Additionally, mental health disorders are emerging as a growing concern.^4^ Addressing this complex and dynamic health landscape necessitates a responsive, integrated and community-orientated health system.

Primary Health Care (PHC), delivered through the district health system (DHS), plays a vital role in responding to the healthcare needs of the population, particularly in underserved and rural areas. The DHS model in South Africa was implemented in 1997 and provides health services through 52 districts and 242 subdistricts.^5^ As of 2024, approximately 84% of South Africa’s 63 million people relied on the public health sector,^6^ underscoring the urgency of ensuring the DHS is staffed appropriately to deliver high-quality care.^7^

Several strategies have been implemented to strengthen PHC and the DHS. Some provinces have adopted a community-oriented primary care (COPC) approach to deliver PHC, integrating primary care with essential public health functions.^8,9^ Across the country, efforts have been made to expand community-based services through the implementation of community health worker teams to provide proactive outreach, improve health outcomes and reduce health inequities.^8^ In addition, the deployment of district clinical specialist teams (DCSTs) has played an important role. These multidisciplinary teams are tasked with enhancing the quality of maternal, neonatal and child health services through training, mentorship and clinical governance support.^10^

In this context, the role of the FP has gained increasing attention. The World Health Organization (WHO) recognises that the most effective PHC systems are those that include medical practitioners with postgraduate training in family medicine.^11^ Global evidence supports the inclusion of FPs in PHC systems, demonstrating that family medicine is a cost-effective approach associated with improved health outcomes, lower healthcare expenditure and high levels of user satisfaction.^12,13,14^ Although the specific competencies and roles of FPs may vary with the context, for example the health needs of communities, the model of care and financial resources available, the core principles of family medicine are aligned with the core functions of primary care: first contact access, comprehensiveness, person-centredness, continuity and coordination.^15^

Building on these core principles, international efforts have sought to define and articulate the FP’s role within diverse PHC systems. The Besrour Centre and World Organization of Family Doctors (WONCA) have advanced a global understanding of family medicine that centres on social accountability, responsiveness to local needs and leadership within team-based care models.^16,17^ Despite this shared foundation, the operationalisation of family medicine differs considerably across health systems. In high-income countries, FPs or vocationally trained general practitioners are often the first point of contact. In contrast, in sub-Saharan Africa (SSA), this function is more commonly fulfilled by nurse practitioners and clinical associates, reflecting the region’s acute physician shortages and differing human resource capacities.^18^ Family physicians may also be located at primary hospitals and provide outreach to the primary care platform. These contextual variations have prompted calls for models of care tailored to each setting’s workforce realities, while still adhering to the core principles of family medicine. As such, the definition and implementation of family medicine must remain flexible, supporting the adaptation of FP roles to local epidemiological, demographic and systemic factors.^15,17^

In South Africa, the primary care workforce is structured around community health workers in community-based services, nurse practitioners in primary care clinics, and generalist doctors without formal postgraduate training in district hospitals or health centres. Historically, the DHS did not include specialists in policy.^5^ However, the 2008 World Health Report cautioned against oversimplified models that relied heavily on unsupported health workers, noting their inadequacy in addressing the complexity of primary care.^11^ Family physicians, who are specifically trained to function within both primary care and district hospital settings, are equipped to enhance the quality of care and reduce unnecessary referrals to higher levels of care.^19^

As South Africa moves towards COPC in support of universal health coverage (UHC), FPs play a critical leadership role in implementing community and facility-based services, community engagement and multisectoral collaboration.^19^ Their specialist training enables them to serve in three key areas, which reflect global trends, namely: as a clinician and consultant to the clinical team; as a capacity builder and clinical trainer; and as a leader of clinical governance.^5,18,20^ These roles position them uniquely to provide high-quality services.

In recognition of the critical role of FPs in strengthening the DHS, the South African Academy of Family Physicians (SAAFP) released a revised national position paper in 2021.^21,22^ This paper responded to the evolving health policy landscape and recommended a minimum target of one FP per district hospital, community health centre (CHC) or subdistrict without a CHC by 2030.^21^ The recommendation offers a practical benchmark for human resource planning and is particularly relevant in the context of major reforms, such as the introduction of national health insurance.^10^ The position paper was also needed to respond to the National Human Resources for Health 2030 strategy, which appeared to misunderstand the role of FPs. This strategy only calculated the number of FPs needed for DCSTs and tertiary hospitals.^3^

South Africa lacks accurate, up-to-date data on the distribution of FPs within its Public Health Care system, making it difficult to monitor the goals outlined in the position paper or policy. Estimates suggest that only 29% of registered FPs work in the public sector,^23^ yet the current number, location within the DHS, and employment characteristics remain poorly documented because of fragmented and unreliable human resource databases.^22^ Existing data sources such as the Health Professions Council of South Africa (HPCSA) register and the Personnel and Salary System (PERSAL) are limited in scope, with the former overestimating active practitioners and the latter known for data inaccuracies.^7^

A recent human resource for health analysis estimated a theoretical supply of 0.16 FPs per 10 000 population in 2019, but this estimate does not disaggregate by facility type, metropole status or geographical distribution.^23^ As a result, there is a critical knowledge gap regarding the real-time availability of FPs within DHS entities. Without this information, it is impossible to assess how far the country is from meeting the 2030 SAAFP target or to engage in evidence-based policy and workforce planning. This study, therefore, addresses a significant gap by providing the first national-level mapping of the prevalence of FPs employed in DHS entities in South Africa. This study aimed to determine the proportion and distribution of DHS entities with a FP in the South African Public Health Care sector. The objectives were to:

- Determine the proportion of DHS entities (district health facilities and subdistricts without a CHC or CDC) that have at least one FP employed.
- Quantify the total number of FPs present across all DHS entities (DCSTs, district health facilities and subdistrict level).
- Evaluate the nature and characteristics of their employment posts.
- Compare the distribution of FPs across subdistricts, districts and provinces, as well as metropolitan versus non-metropolitan districts and district facility type.

## Research methods and design

### Study design

This study employed a descriptive, cross-sectional design, with quantitative data collected for the period May to October 2024.

### Setting

The study was conducted across all nine provinces of South Africa, within the public sector, specifically targeting the DHS. The DHS model is a nationally endorsed health delivery system that serves as the foundation of South Africa’s hierarchical healthcare system. There are 52 geographically defined and contiguous districts (44 district municipalities and eight metropolitan municipalities) with 242 subdistricts, each responsible for providing health services to well-defined populations. Health services include DCSTs at a district level, a range of primary care facilities (smaller clinics, community day centres [CDCs], community health centres [CHCs]) and district hospitals.

Primary care facilities are predominantly nurse-led and managed. The CDCs typically operate for eight hours per day, whereas CHCs provide 24-h services, often including midwife-led maternity care and basic emergency services.^24^ Medical officers are usually found at CDCs and CHCs and may visit clinics on a regular basis. District hospitals are classified as small, medium or large, and deliver both outpatient and inpatient services. These hospitals play a critical role in supporting primary care facilities and serve as referral centres for patients requiring access to a higher level of care.^24^

Family physicians are trained to serve within the DHS, typically based at larger facilities such as district hospitals, CHCs or CDCs, from which they provide clinical care and outreach to smaller peripheral clinics. At the broader district level, they may also form part of DCSTs, contributing to clinical governance and health system strengthening. In some settings, FPs are also deployed in regional and tertiary hospitals, where they work in departments of family medicine or emergency centres, alongside other specialist disciplines.^22^

The speciality was promulgated in 2007, when the HPCSA and the national government recognised family medicine as a distinct medical speciality.^15^ Many FPs were grandfathered onto the register when it was first opened, but now a FP must complete clinical training (including a research assignment) and the Fellowship examination to register.^23^

### Study population and sampling strategy

The study employed a complete census approach, collecting data from all DHS entities rather than using sampling. All 52 districts were included in the study, encompassing DCSTs, target facilities and subdistricts without a CHC or CDC. Target facilities, as defined in this study, included CHCs and district hospitals in alignment with the SAAFP position paper recommendations. The authors also included CDCs to account for provincial variation in facility classification – specifically in the Western Cape and Gauteng – where CDCs are differentiated from CHCs. This inclusion aimed to reduce classification error and ensure consistency across provinces.

The study excluded clinics, private healthcare facilities and public health sector institutions situated at higher levels of care, including regional, tertiary, central and specialised hospitals (e.g. psychiatric and tuberculosis hospitals), as well as non-clinical managerial or academic posts in district offices or universities. These exclusions were based on the study’s focus on DHS facilities specifically referenced in the SAAFP position paper, which advocates for the employment of FPs at district hospitals, CHCs and subdistricts without a CHC. While it is acknowledged that FPs based outside the DHS may contribute to service delivery through outreach or support, this study aimed to map the distribution and supply of FP posts located *within* the DHS, in alignment with the targets proposed in the position paper.

Thus, the study included all FPs, registered with the HPCSA, working in the DHS in a DCST, target facility or subdistrict. They could be in various employment positions, including FP posts, medical officer posts and facility-based managerial posts. Additionally, although many Cuban-trained FPs work in South African healthcare facilities, this study excluded those not registered with the HPCSA as FPs.

Survey respondents were family medicine academics affiliated with one of South Africa’s ten universities offering postgraduate training in family medicine. The Education and Training Committee (ETC) of the SAAFP identified these individuals for their intimate knowledge of the DHS footprint within their university’s catchment area, as shown in Table 1. Two respondents from each university were paired to cross-check and jointly complete the survey, improving the reliability of the data.

**TABLE 1:** South African university departments of family medicine, with their associated provincial footprints.

Universities with departments of family medicine	Geographical footprint by province
University of Cape Town	Western Cape
Stellenbosch University	Western Cape and Northern Cape
University of the Witwatersrand	Gauteng, North West
University of Pretoria	Gauteng and Mpumalanga
University of Limpopo	Limpopo
Sefako Makgatho Health Sciences University	Gauteng, North West
University of KwaZulu-Natal	KwaZulu-Natal
University of the Free State	Free State and Northern Cape
Walter Sisulu University	Eastern Cape
Nelson Mandela Metropolitan University	Eastern Cape

### Data collection

The national District Health Information System (DHIS) provided a current database of DHS entities, comprising 52 districts, 242 subdistricts and 607 target facilities. Based on this, quantitative data on the presence of a FP, the number of FPs and employment characteristics were collected for each DCST, target facility and relevant subdistrict. The data were entered directly into a custom-developed, structured Microsoft Excel spreadsheet survey tool. The tool’s terminology was clearly defined to standardise responses. A FP was described as a family medicine specialist registered with the HPCSA on the post-2007 specialist register.

Before implementation, the tool was piloted with respondents from one of the universities to ensure clarity. Minor adjustments were made following the pilot to improve tool usability. The survey tool was distributed via email to identified respondents, along with an information leaflet and consent form. Respondents had 1 month to complete the tool. Non-responders were followed up by phone, and in cases of non-participation, alternative respondents were identified by the research team or suggested by local contacts. All respondents were proficient in English, and no language barriers were anticipated or reported.

### Data analysis

Data obtained from each university were reviewed, checked for errors or omissions and consolidated into a single Microsoft Excel spreadsheet. Subsequent summary statistics and choropleth maps created through QGIS Geographic Information System (version 3.42) were used as descriptive statistical methods. Inferential statistical analyses were conducted using R statistical software (version 4.4.3).^25,26^

The data were analysed in relation to the study objectives: firstly, to determine the proportion of DHS entities with at least one FP employed; and secondly, to quantify the total number of FPs across all DHS entities during the study period.

The analysis commenced with a descriptive exploration of the dependent variables – defined as the presence (binary) and count (numerical) of FPs at each assessed level – and the independent variables, which included both categorical variables (e.g. province, district, subdistrict, hospital status, metropole status) and numerical variables (e.g. number of facilities per subdistrict). Categorical data were summarised using frequencies and proportions.

For the inferential analysis, the focus was to examine whether specific characteristics were associated with the likelihood of having a FP employed. Specifically, the analysis assessed:

- The type of target facility (i.e. CDC, CHC or district hospital).
- The metropolitan or non-metropolitan location of the target facility.
- The number of target facilities in a subdistrict.

Inferential statistical analyses were performed at the target facility level, employing both crude and adjusted odds ratio (OR) calculations derived from logistic mixed-effects regression models. These variables were examined to determine their association with the likelihood of employing at least one FP in different types of facilities and metropolitan locations. Covariates (district hospital status and metropole status) included as fixed effects in the models were decided *a priori* based on expert opinion in the subject matter. A significance threshold of *p* < 0.05 was applied consistently across all statistical tests.

### Ethical considerations

This study received approval from the Health Research Ethics Committee of the University of Cape Town (reference number: 911/2023). Collaboration with the national ETC of the SAAFP was undertaken to support the selection and engagement of survey respondents. Participants were informed of the purpose and rationale of the research, and written informed consent was obtained prior to participation in data collection.

## Results

**FIGURE 1:**
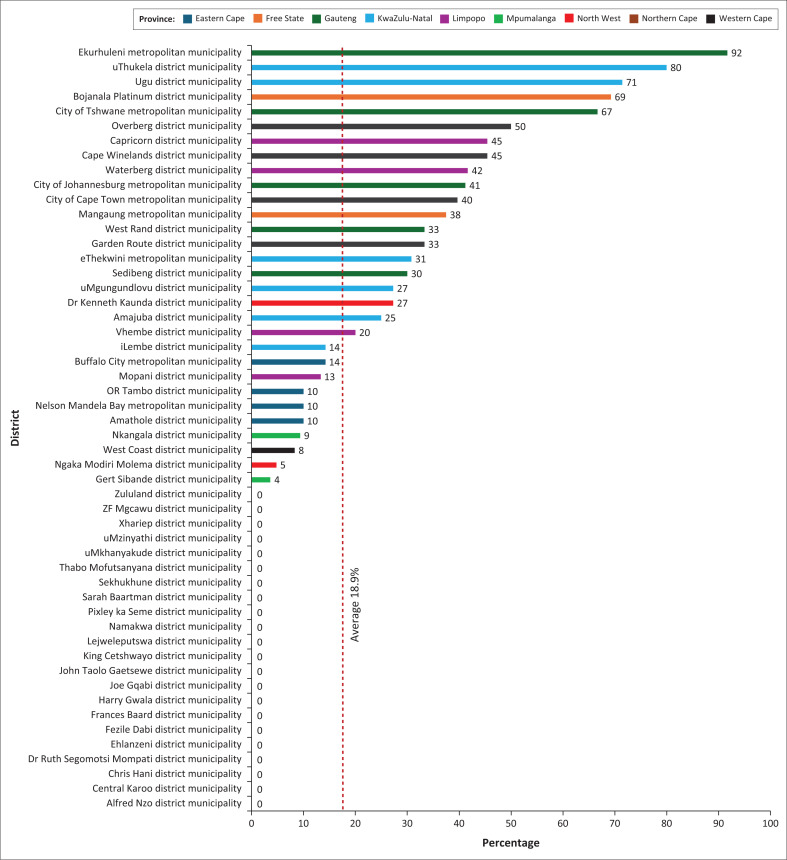
The percentage of district health entities with a family physician by district and province in South Africa.

Among the 52 districts, 34 had an operational DCST, and data were available for 605 of the 607 target facilities in the DHIS database, with two facilities – SAWAS Memorial Hospital and Settlers Day Hospital in the Eastern Cape – excluded because respondents were unsure of their status. Of the 242 subdistricts, 95 lacked a community health or day centre. The results are presented as the proportion of DHS entities with at least one FP employed, presented in Table 2 and Figure 1, as well as the total number of FPs present across all DHS entities, presented in Table 3, Table 4 and Figure 3, during the study period.

**TABLE 2:** The provincial distribution of family physicians (*N* = 132 of 700) within district health system entities in South Africa.

Province	Total			Subdistricts without CHC or CDC			CHC or CDC			District hospitals		
	*n*	*N*	%	*n*	*N*	%	*n*	*N*	%	*n*	*N*	%
Gauteng	33	60	55.0	2	5	40.0	23	43	53.5	8	12	66.7
Western Cape	40	116	34.5	3	12	25.0	20	71	28.2	17	33	51.5
Limpopo	15	64	23.4	3	8	37.5	2	26	7.7	10	30	33.3
North West	13	62	21.0	0	1	0.0	9	48	18.8	4	13	30.8
KwaZulu-Natal	18	93	19.4	7	29	24.1	1	23	4.3	10	41	24.4
Free State	3	50	6.0	1	15	6.7	0	10	0.0	2	25	8.0
Eastern Cape	6	121	5.0	0	16	0.0	1	40	2.5	5	65	7.7
Mpumalanga	4	84	4.8	1	3	33.3	0	58	0.0	3	23	13.0
Northern Cape	0	50	0.0	0	6	0.0	0	32	0.0	0	12	0.0
**Total**	**132**	**700**	**18.9**	**17**	**95**	**17.9**	**56**	**351**	**16.0**	**59**	**254**	**23.2**

CHC, community health centre, CDC, community day centre.

**TABLE 3:** Total number of family physicians per district health system entity across South African provinces.

Province	Total	District clinical specialist teams	Subdistricts	Target facility
	*n*	*n*	*n*	*n*
Total	214	23	28	163
Western Cape	71	-	7	64
Gauteng	47	4	5	38
KwaZulu-Natal	24	4	5	15
North West	20	3	3	14
Limpopo	18	-	5	13
Eastern Cape	15	5	2	8
Free State	9	5	1	3
Mpumalanga	8	-	0	8
Northern Cape	2	2	0	0

**TABLE 4:** Employment characteristics of family physicians employed in the district health system entities in South Africa, stratified by the location of the employment post.

Characteristic	Total FPs (*N* = 214)		FPs in DCSTs (*N* = 23)		FPs in Target facilities (*N* = 163)		FPs in Subdistricts (*N* = 28)	
	*n*	%	*n*	%	*n*	%	*n*	%
**Employment post**								
Family physician	168	78.5	21	91.3	124	76.1	23	82.2
Medical officer	25	11.7	0	0.0	23	14.1	2	7.1
Manager	16	7.5	0	0.0	14	8.6	2	7.1
Uncertain	5	2.3	2	8.7	2	1.2	1	3.6
**Nature of post**								
Fulltime	206	96.3	23	100	157	96.3	26	92.9
Sessional	8	3.7	0	0.0	6	3.7	2	7.1
Uncertain	0	0.0	0	0.0	0	0.0	0	0.0
**Type of post**								
Joint Appointed	87	40.7	9	39.1	67	41.1	11	39.3
Non-joint Appointed	124	57.9	13	56.5	94	57.7	17	60.7
Uncertain	3	1.4	1	4.3	2	1.2	0	0.0

FPs, family physicians; DCSTs, district clinical specialist teams.

Table 2 presents a summary of the provincial distribution of FPs within DHS entities in South Africa. Of the 700 DHS entities assessed – including 605 target facilities and the 95 subdistricts without a CHC or CDC – the overall prevalence of a FP was 18.9%. This can be disaggregated into 17.9% of relevant subdistricts having a FP, 16.0% of CHCs or CDCs and 23.2% of district hospitals. Across provinces, Gauteng (55.0%) and Western Cape (34.5%) showed the highest proportions of target facilities with a FP, while the Northern Cape had no recorded FP in any DHS entity.

Figure 1 presents the percentage of DHS entities – defined in alignment with the SAAFP position paper – that had at least one FP across all 52 districts in South Africa. The figure highlights marked variation in coverage both within and between provinces. A total of 22 districts reported no FPs, including all districts in the Northern Cape. Several districts in the Eastern Cape, KwaZulu-Natal, Free State, Mpumalanga, Limpopo and North West also recorded no coverage. In contrast, higher percentages were observed in other districts, with 92% coverage in the Ekurhuleni metropolitan municipality (Gauteng), 80% coverage in UThukela (KwaZulu-Natal), 71% coverage in Ugu (KwaZulu-Natal) and 69% coverage in the Bojanala Platinum district municipality (North West). In total, 20 of the 52 districts had a prevalence of FPs above the national average of 18.9%.

**FIGURE 2:**
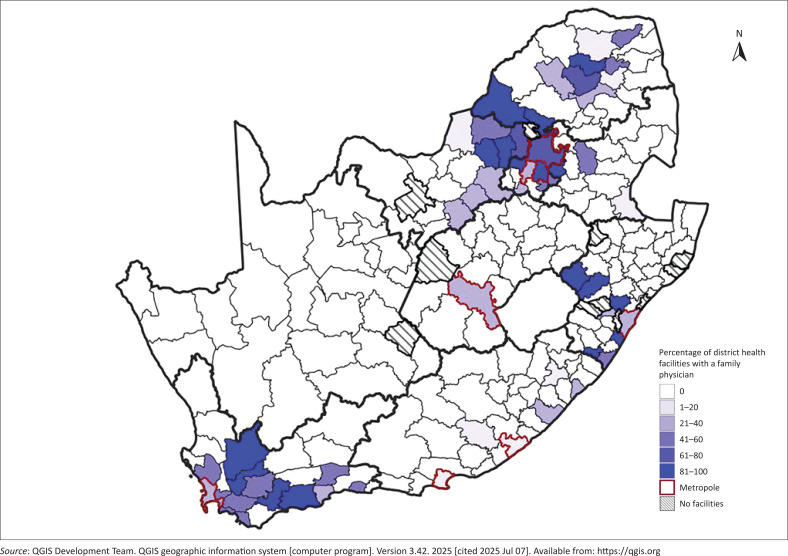
The percentage of district health target facilities with a family physician by subdistrict in South Africa.

Figure 2 illustrates the percentage of target facilities within each subdistrict that had at least one FP employed during the study period. The data show that 69.0% (167 of 242) of subdistricts had no FP employed at any of their target facilities. These subdistricts were primarily located in the Eastern Cape, Free State, KwaZulu-Natal, Mpumalanga, North West and Northern Cape. Only 62 subdistricts recorded coverage levels above the national average of 18.9%. The figure also indicates that certain provinces and metropolitan areas had a greater concentration of FPs at target facilities. A total of 26 subdistricts demonstrated full (100%) coverage across all target facilities, with Gauteng accounting for 10 of these. Other subdistricts with complete coverage included those in Bojanala Platinum (Kgetlengrivier and Rustenburg), Cape Winelands (Breede Valley and Witzenberg) and multiple subdistricts within the Ekurhuleni, Johannesburg and Tshwane metropolitan municipalities. Overall, for every one target facility that had a FP, there were 4.35 target facilities that did not.

Table 3 presents the number of FPs employed across DHS entities by province. A total of 214 FPs were recorded as employed within DHS entities. The Western Cape accounted for the highest number (71), followed by Gauteng (47) and KwaZulu-Natal (23). Each of the remaining provinces recorded 20 or fewer FPs. Of the 34 DCSTs, 22 had one FP, with one team in the Free State employing two. Across all 242 subdistricts, 28 FPs were employed at the subdistrict level in the subdistrict offices, outside of a DCST or target facility. A total of 163 FPs were employed across 115 of the 605 target facilities. The provincial distribution is further detailed by DCST, subdistrict and target facility categories.

**FIGURE 3:**
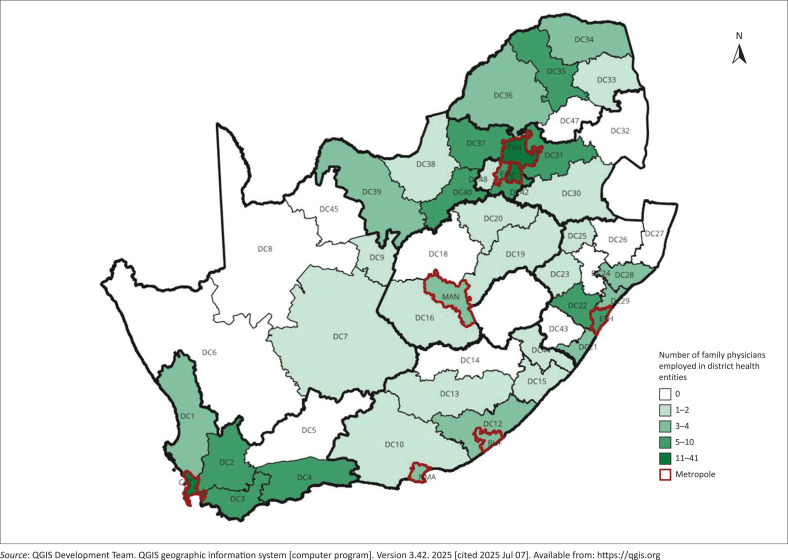
The total number of family physicians in district health system entities by district in South Africa.

Figure 3 displays the total number of FPs by district in DHS entities. This shows that 12 of 52 districts in South Africa have no FPs employed. Additionally, it shows that three metropolitan municipalities have the highest number of FPs: Cape Town, Tshwane and Ekurhuleni, with 41, 18 and 13 FPs, respectively. The figure highlights variation across both urban and rural districts and across all provinces.

Table 4 presents a summary of the employment characteristics of the 214 FPs working in the DHS entities, stratified by post location. Within these entities, the majority (76.2%) of FPs were employed in target facilities, with 13.1% based at the subdistrict level and 10.7% in DCSTs. Most FPs (78.5%) held specialist FP posts, while 11.7% were employed in medical officer posts and 7.5% held managerial roles. Most posts (96.3%) were full-time, including all DCST positions, while sessional appointments accounted for 3.7%. Additionally, 40.7% of FPs were in joint university-affiliated posts, a pattern that was consistent across post locations.

In a multiple variable analysis, district hospitals were more likely to have a FP than primary care facilities (OR 8.5; 95% CI: 4.1–17.4, *p* < 0.001), and metropole-based facilities were more likely than non-metropolitan districts (OR 20.9; 95% CI: 4.0–109.4, *p* < 0.001). Target facilities that were both district hospitals and situated in metropolitan areas were substantially more likely to have a FP (OR 177.9; 95% CI 24.8–1273.7, *p* < 0.001) compared to that of a facility with neither.

## Discussion

South Africa has achieved 18.9% of the target set by the SAAFP for deployment of FPs. There is enormous variability between provinces, with Gauteng achieving 55% coverage and the Northern Cape 0% coverage. The Western Cape and Limpopo also had above-average coverage. FPs were more likely to be employed in a district hospital and a metropolitan district than in primary care or a rural area. Approximately 1 in 10 were employed in medical officer and not FP posts, and almost all were full-time positions. A substantial number (41%) were joint staff with universities.

### Key findings

The study confirms that while progress has been made in the deployment of FPs, the current supply and distribution of FPs within South Africa’s DHS remain below the recommendations of the 2021 SAAFP position paper. With 568 DHS entities lacking a FP during the study period, the health system is unlikely to realise this recommendation by 2030. Despite the formal recognition of family medicine as a speciality in 2007 and the creation of training programmes, the DHS remains under-resourced in terms of FPs. This shortfall is compounded by human resource policies that have historically misunderstood the role of FPs and based workforce projections primarily on the needs of DCSTs and tertiary hospitals, rather than the broader requirements of the DHS – highlighting the need for more contextually informed workforce planning.^10,19,21^

Limited FP coverage affects service delivery across multiple levels of the health system. However, existing research has not consistently demonstrated the impact of FPs in primary care facilities, and further studies are needed to better understand their role and effectiveness in these settings.^27^ In primary care settings, insufficient availability of FPs likely undermines clinical oversight, care coordination and the management of complex conditions, which may contribute to increased referrals and added strain on higher levels of care.^19,21^ Additionally, the constrained impact of FPs in this context undermines national efforts to strengthen COPC. In district hospitals without FPs, significant skills gaps and inadequate supervision and clinical oversight may contribute to system failures, resulting in increased medico-legal claims and a lack of improvement in maternal health outcomes.^10,19^ In both settings, FPs drive improvements in quality of care and patient safety.

The multivariable analysis indicates that district hospitals were 8.5 times more likely to have a FP employed compared to primary care facilities, when controlling for the confounding effect of metropole status. This statistically significant association (*p* < 0.001) suggests a strong institutional preference for employing FPs within district hospitals. The wide confidence interval (95% CI: 4.1–17.4) reflects some variability across settings, but the lower bound remains well above 1, reinforcing the robustness of this finding.

The increased likelihood of FP employment at district hospitals may reflect institutional prioritisation of posts within these settings, rather than differences in the complexity of care required across the DHS. In practice, patients with complex and undifferentiated conditions present across all levels of care, including primary care facilities, where early diagnosis, continuity and coordination of care are critical. District hospitals in South Africa provide a significant portion of inpatient care, accounting for 35.9% of all hospital beds nationally^28^ yet only 23.2% of these hospitals reported having a FP. While district hospitals play an important role in managing admitted patients and supporting referral pathways to regional hospitals, FPs are equally positioned to contribute across the continuum of care, including at primary care level. Their broad training and clinical competencies enable them to support the management of complex conditions, strengthen clinical decision-making, and enhance coordination of care, which may reduce unnecessary referrals to higher levels of care and improve system efficiency and access.^18,20,29^ However, the current clustering of FPs within district hospitals may contribute to service gaps in clinical support and governance at primary care level, particularly in rural subdistricts.

Similarly, the multivariable analysis indicates that facilities located within metropolitan districts were 20.9 times more likely to have a FP employed compared to those in non-metropolitan areas, when controlling for the confounding effect of facility type. This statistically significant association (*p* < 0.001) indicates a strong geographic concentration of FP posts in metropolitan areas. This association between FP presence and metropolitan status is consistent with longstanding evidence of geographic maldistribution of healthcare professionals and undersupply of specialists in rural and underserved areas, with urban areas offering more attractive working conditions and infrastructure.^3,18^ Proximity to training institutions also appears to facilitate FP deployment, as graduates often remain in areas where they train.^19,21^ Rural and under-resourced areas with minimal FP presence face challenges because of limited infrastructure, geographic isolation and weaker support systems.^22,23^

The marked provincial and intra-provincial variation in deployment poses challenges for the discipline. Provinces such as the Western Cape and Gauteng have comparatively higher coverage, while others – particularly the Northern Cape, Eastern Cape and Mpumalanga – report minimal or no FP presence in many districts. At the same time, the relatively higher coverage observed in provinces such as Gauteng and the Western Cape demonstrates that more extensive FP deployment is achievable within the current health system. Several factors may underpin this variability, including differences in leadership, policy formulation and implementation, as well as advocacy for family medicine and other resource allocation strategies.

The results reveal a clear pattern of concentration, with FPs predominantly located in metropolitan district hospitals and clustered within certain provinces and districts, while rural and primary care facilities remain markedly underrepresented. This uneven distribution underscores systemic imbalances in workforce planning within the DHS, where factors such as clinical complexity and infrastructure availability appear to influence FP deployment more strongly than population health needs or equitable service accessibility. Moreover, provincial structural differences – including the disproportionately high number of district hospitals in provinces such as the Eastern Cape, compared to more urbanised provinces like Gauteng, as well as the absence of DCSTs in the Western Cape and Limpopo – may further contribute to the observed variability. These contextual differences highlight the importance of interpreting FP distribution patterns within the broader organisational and policy environments of each province.

With regard to leadership, consistent and stable governance may facilitate effective allocation and sustained resourcing within provincial health systems, as reflected in the Western Cape’s strong performance in FP employment. However, the relationship between leadership stability and workforce outcomes does not appear to be linear. Despite frequent turnover in senior leadership positions, Gauteng – alongside the Western Cape – continues to perform comparatively well in employing FPs, although the specific drivers of this variation cannot be determined from this study. While leadership and governance may play a role in shaping workforce outcomes, this relationship is likely to be complex and context dependent. Other factors, such as provincial prioritisation of FP posts, alignment with national policy, and local advocacy for the discipline, may also influence the creation and sustainability of FP posts.^18,20^ Additionally, support at district and facility level has been associated with improved recruitment and retention of FPs.^19^ These findings highlight the need for further research to better understand the factors influencing FP deployment across different provincial contexts.

The inability to showcase the added value of FPs at scale in some provinces may, in turn, affect future investments in posts and training programmes, creating a feedback loop that slows the development and contribution of the discipline.^19^ In provinces where FPs are absent or underutilised, policymakers and facility managers may be less familiar with their scope of practice and potential contributions, further weakening advocacy for expansion. Addressing this variability and improving FP coverage is therefore not only essential to strengthening the DHS – it is also critical to building the visibility, recognition and long-term sustainability of the family medicine discipline within South Africa’s health system.

The high proportion of FPs in full-time specialist posts is encouraging, suggesting increasing recognition and integration of the role within the DHS. The relatively high percentage (40.7%) holding joint university appointments may reflect successful university advocacy efforts related to postgraduate training programmes.

International evidence supports the integration of FPs into primary care teams for improved health outcomes, quality of care and system resilience, particularly in low- and middle-income settings.^1,3,30^ Within the South African context, FPs contribute as expert generalists through clinical care, clinical governance and capacity-building, strengthening continuity and coordination of care across the health system.^21,22,31^ Despite this recognised potential, this study’s findings highlight that FPs remain underutilised across the DHS in South Africa. Strengthening the integration of FPs into primary care teams – especially in underserved areas – may be critical to consolidating a resilient and responsive model of care delivery aligned with national health policy goals.

### Strengths and limitations

A key strength of this study is its national scope and the use of an original, validated data collection tool aligned with the national facility database. The survey achieved a completion rate of 99.7%, due to minor missing data and provides the first comprehensive mapping of FP presence across facility types and provinces in the DHS.

However, limitations include the reliance on self-reporting by senior family medicine academics affiliated with one of South Africa’s ten universities offering postgraduate training in family medicine, which may introduce reporting bias or inaccuracies; however, this was mitigated by pairing respondents. Notably, alternative data sources such as the HPCSA register and the PERSAL system have been shown to be less accurate or outdated for identifying the current deployment of FPs, thereby reinforcing the value of a targeted academic-led reporting strategy for this analysis.

Family physicians in the Public Health Care sector are mainly employed within the DHS; however, this study recognises that the location of employment of FPs varies greatly between provinces because of differing human resources policies, and thus despite engaging in outreach to districts facilities, many FPs employed at tertiary, provincial and regional hospitals were excluded from this dataset, particularly in Free State, Northern Cape and KwaZulu-Natal.

### Recommendations

This study provides the first national mapping of FP deployment across South Africa’s DHS and identifies substantial gaps in both coverage and distribution. Based on these findings, a limited number of evidence-aligned recommendations can be made.

Firstly, the overall low coverage (18.9%) indicates a substantial shortfall relative to existing national benchmarks, with 568 DHS entities lacking a FP. This supports the need for strengthened workforce planning processes that explicitly include FPs within DHS staffing models. Current planning approaches should be reviewed to ensure that they accurately reflect the distributional requirements of the DHS rather than focusing predominantly on higher levels of care or specific programme structures. Furthermore, these findings point to the importance of sustained advocacy efforts, informed by existing policy guidance such as the 2025 SAAFP policy brief,^32^ to facilitate the integration of FPs into national and provincial workforce planning frameworks and to support the effective translation of policy into practice.

Secondly, the marked inequitable distribution – particularly the strong association with district hospitals (OR 8.5) and metropolitan districts (OR 20.9) – suggests that current deployment is influenced more by facility type and geographic context than by equitable population need. Workforce planning processes should therefore incorporate mechanisms to monitor and address geographic and facility-level disparities in FP deployment.

Thirdly, the finding that a proportion of FPs (11.7%) are employed in non-specialist (medical officer) posts likely reflects a shortage of available specialist FP posts within the DHS, resulting in trained specialists occupying roles not aligned with their level of training. This misalignment suggests a need to expand and better align specialist FP posts with the existing workforce to optimise the contribution of FPs.

Finally, this study highlights the importance of accurate, up-to-date human resource data. Strengthening routine data systems to capture FP deployment at facility level would enable ongoing monitoring of workforce distribution and support evidence-based planning.

Further research is needed to assess the impact of FP deployment on service delivery, clinical outcomes and health system performance, particularly in primary care and rural settings, where current coverage is lowest.

By mapping current gaps, this study lays the groundwork for a more equitable, efficient and clinically effective DHS – one that reflects the global and national vision for Primary Health Care transformation.

## Conclusion

This national mapping study assessed the presence and distribution of FPs within South Africa’s DHS and demonstrates both the progress made and the remaining gaps in the deployment of FPs. Firstly, the study found that only 18.9% of DHS entities had at least one FP employed; this shows a growing national footprint, but also highlights a substantial gap relative to national policy and professional body recommendations. Secondly, a total of 214 FPs were recorded as employed across the DHS during the study period, with the majority based in metropolitan areas and district hospitals. This represents a substantial national cohort of FPs working within the DHS, providing a foundation for further expansion. At the same time, these findings underscore the uneven and context-dependent nature of FP deployment, with implications for equity in service delivery and for the discipline’s capacity to demonstrate its value. Achieving broader and more equitable coverage will require coordinated efforts in human resource planning, training and policy implementation.
